# Identification and validation of STAT4 as a prognostic biomarker in acute myeloid leukemia

**DOI:** 10.1042/BSR20231720

**Published:** 2024-02-12

**Authors:** Chenyu Li, Jingyuan Zhao, Bingyu Kang, Shuai Li, Jingya Tang, Deshi Dong, Yanwei Chen

**Affiliations:** 1 Department of Pharmacy, The First Affiliated Hospital of Dalian Medical University, Dalian, China; 2 Stem Cell Clinical Research Center, The First Affiliated Hospital of Dalian Medical University, Dalian, China; College of Pharmacy, Dalian Medical University, Dalian, China3

**Keywords:** AML, prognostic biomarker, STAT4, transcription factor, Treg

## Abstract

Acute myelogenous leukemia (AML) is a common malignancy and is supposed to have the ability to escape host immune surveillance. The present study aimed to identify key genes in AML that may affect tumor immunity and to provide prognosis biomarkers of AML. The Cancer Genome Atlas (TCGA) dataset was screened for transcription factors (TFs) involved in immunity and influencing survival, combining Gene Expression Omnibus (GEO) data to validate the impact on patient survival. A prognostic signature was established using four transcription factors, and these genes play an important role in the immune system, with higher regulatory T cell (Treg) scores in high-risk patients compared with the low-risk group. Analysis of individual genes showed that STAT4 and Treg are closely related, which may be due to STAT4 transcribing related genes that affect immunity. STAT4 expression was positively correlated with the proportion of abnormal cells and promoted AML recurrence as verified by AML clinical patient samples. In addition, silencing of STAT4 significantly slowed down the proliferation capacity of HL60 cells. In conclusion, these findings suggest that STAT4 may be a potential biomarker for AML prognosis. As a key gene affecting the prognosis of AML patients, STAT4 has the potential to be a candidate diagnostic and prognostic biomarker for AML.

## Introduction

Acute myeloid leukemia (AML) is a malignant clonal disease caused by myeloid or hematopoietic progenitor cells. It is characterized by rapid progression, a short natural course, and a poor prognosis [1]. AML progresses rapidly and most patients eventually become resistant to treatment or relapse and develop relapsed or refractory AML, with a 5-year survival rate of only 29.5% and no significant improvement in 5-year overall survival in patients older than 65 years [2].

Immunotherapy is one of the most promising approaches for the treatment of tumors [3], and the interaction of the immune system with AML cells offers the possibility of immunotherapy for AML [4]. Immunotherapeutic approaches to AML include antibody-mediated therapies, checkpoint inhibitors and T-cell retargeting therapies, but the development of immunotherapy is still in its early stages due to the lack of unique targets and immune-mediated adverse events observed in studies [5]. Given the importance of immunotherapy in the overall treatment of AML, especially for drug-resistant patients [6], the discovery of new immunotherapeutic markers is urgent.

Regulatory T cells (Treg) are a subpopulation of T cells with immunosuppressive regulatory immune functions that antagonize effector T cells and maintain immune homeostasis [7,8]. In recent years, numerous studies have shown that Treg cells can impair immune surveillance of tumors by the immune system, suppress the responses of anti-tumor immune, and promote tumor growth [9,10]. Therefore, Treg cells are considered to be an important target for tumor immunotherapy. Exploring the role of Treg cells in AML and gaining insight into the relationship between Treg cells and leukemic immune response as well as prognosis is essential to improve the therapeutic effect of AML.

In the present study, we obtained gene expression profiles of AML patients from the TCGA database and then constructed a prognostic model based on transcription factors. Immune scores were calculated based on the prognostic model, the genes that exert an influence on tumor immunity were subsequently identified. The role of the gene was validated by analyzing AML clinical patients.

## Materials and methods

### Data collection and identification of differentially expressed genes (DEGs)

**Figure 1:**
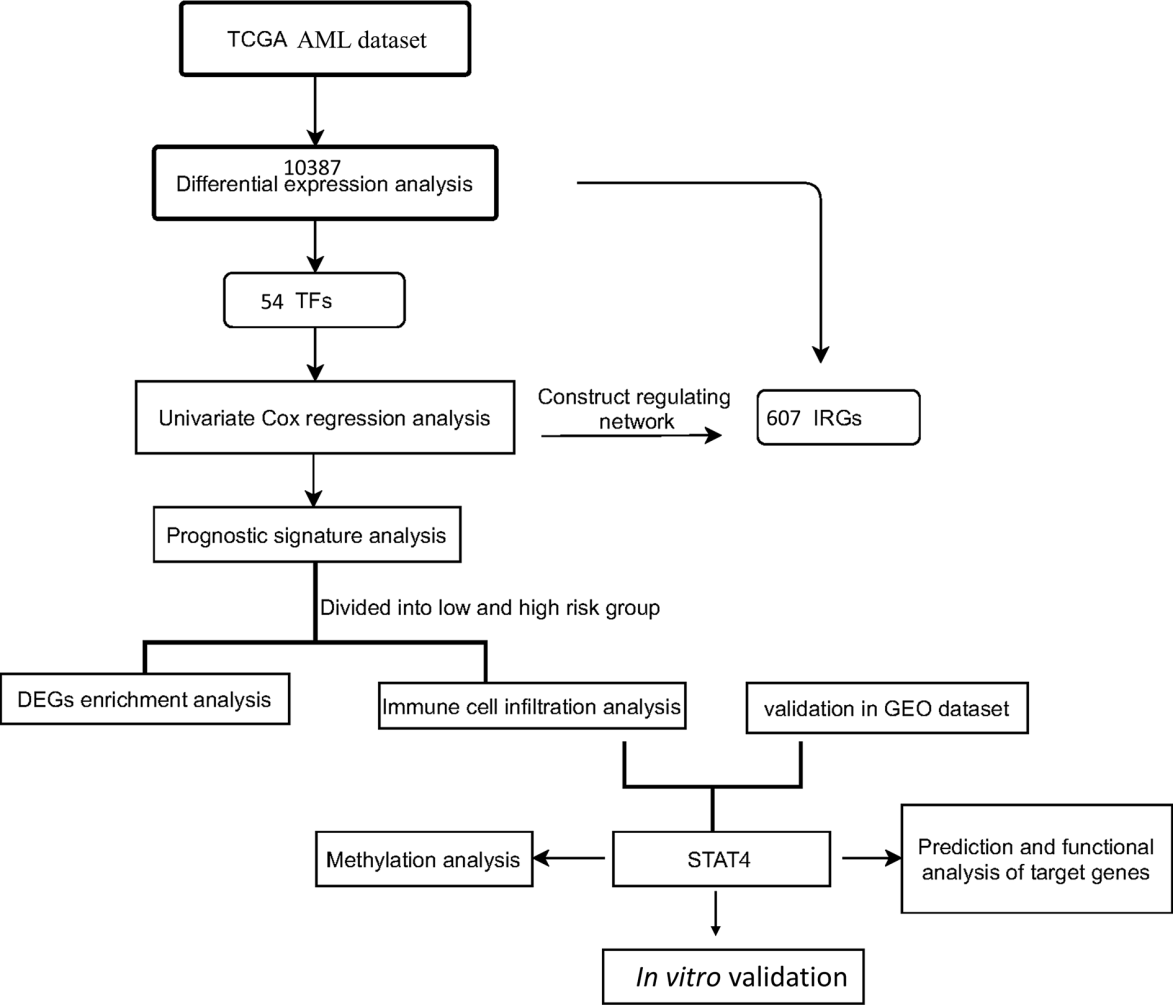
Flowchart of study

A schematic representation of our study outline is shown in Figure 1. Gene expression profiling interactive analysis (GEPIA2) web server (http://gepia2.cancer-pku.cn/#analysis) was used to obtain expression difference between AML and normal tissues of the GTEx database. The genes which meet the following criteria are defined as DEGs: the cut‐off value |log 2‐fold‐change [FC]| > 1 and *P-*value< 0.05. The AML RNA-seq transcriptome profiles and related clinical information were downloaded from The Cancer Genome Atlas (TCGA). The immune-related genes (IRGs) were identified through the ImmPort database (https://www.immport.org/shared/) and the transcription factor (TF) set was downloaded from Cistrome Cancer (http://cistrome.org/CistromeCancer/).

### Identification and validation of TF-IRG regulatory network

Univariate cox regressions were used to evaluate the relationships between the TFs and the patient’s overall survival (OS). TFs with *P*<0.05 and Hazard Ratio (HR) > 1 were screened for further analysis. After that, TFs and IRGs were carried out for further protein-protein interaction (PPI) network construction. PPI network was constructed by the STRING website and Cytoscape to elucidate potential interactions between TFs and IRGs.

### Prognostic signature construction

The least absolute shrinkage and selection operator (LASSO) analysis by using R software was performed to select optimal prognostic TFs. The multivariate Cox regression analysis was used to perform the risk model. The Risk score (RS)formula is: Risk score (RS) = Σ gene expression ×coefficient, based on the median value, the RS values were divided into high-risk and low-risk groups. The patients were divided into high-risk and low-risk groups based on the median risk score as the cutoff value. To assess the performance of our signatures, the ‘survival receiver operating characteristic (ROC)’ package was used to generate ROC curves at 1, 3 and 5 years, and the corresponding time-dependent area under the curves (AUCs) was calculated simultaneously. The performance of TFs-based signature was assessed using Kaplan–Meier analysis.

### Enrichment analysis of DEGs between high-risk and low-risk groups

Expression profiles were compared between high-risk and low-risk groups to identify DEGs by using the R software limma package. Gene Ontology (GO) and Kyoto Encyclopedia of Genes and Genomes (KEGG) pathway enrichment analyses of the DEGs were performed using the ‘ClusterProfiler’ R package.

### Tumor-infiltrating immune cell profile

CIBERSORT was used to estimate the immune cell in filtration fraction and analyze the difference between high-risk and low-risk groups. Pearson correlation was calculated between the proportions of immune cells and the target gene expression.

### Validation of the key genes based on TCGA and GEO

GEPIA was used to verify the survival and expression patterns. Three external GEO AML cohorts-GSE5122 and GSE12417 were used as validation sets by using the PrognoScan database (http://dna00.bio.kyutech.ac.jp/PrognoScan/).

### Validation and analysis of target gene

The ChEA3 (https://maayanlab.cloud/chea3/) was used to estimate the target of TF. The OS and methylation status of the gene were analyzed by Gene Set Cancer Analysis (GSCA) (http://bioinfo.life.hust.edu.cn/GSCA/#/) which is a tumor genome analysis platform that integrates genomic data from the TCGA library and normal tissue data from GTEX; For genomic analysis in a unified data analysis process.

### Validation of the STAT4 in clinical tissue samples by real-time PCR

A total of 17 AML patient and 10 healthy donors blood samples were removed from the First Affiliated Hospital of Dalian Medical University (Liaoning, China) from 2022 to 2023. The samples have been approved by the Ethics Committee of the First Affiliated Hospital of Dalian Medical University, and all subjects provided written informed consent. The STAT4 gene mRNA expression was assayed by real-time PCR. Total RNA was extracted with TRIzol reagent (Thermo Fisher, Cat # 15596026), followed by reverse transcription of cDNA with a kit (Thermo Fisher, Cat # M16325). Real-time PCR was performed using the SYBR Green PCR Master Mix (Thermo Fisher, Cat # 4309155). PCR amplification regime of 95°C for 1 min, 40 cycles of 95°C for 10 s, 60°C for 10 s, 72°C for 15 s, and a final elongation of 72°C for 30 s was used. The qPCR primer sequences used were listed as follows: STAT4 forward: 5′-TGTTGGCCCAATGGATTGAAA′ and reverse: 5′-GGAAACACGACCTAACTGTTCAT′, GAPDH forward: 5′-ACAACTTTGGTATCGTGGAAGG-3′ and reverse: 5′- GCCATCACGCCACAGTTTC-3′. The amplicon size of productions is 119 bp.

### STAT4 knockdown

The short hairpin RNAs against STAT4 and its corresponding nonspecific vectors were designed and constructed by Genechem Company (Shanghai, China). Lipofectamine 3000 transfection reagent (Thermo Fisher Scientific, U.S.A.) was used to transfect HL60 cells with several plasmids in accordance with the owner’s manual. Cells were harvested 48 h after transfection in preparation for the upcoming tests.

### Western blotting

To prepare total protein, the cells were lysed in RIPA buffer supplemented with 1% PMSF on ice. Extracted protein samples were analyzed through 12.5% sodium dodecyl sulfate-polyacrylamide gel electrophoresis (SDS-PAGE) and were electro-transferred onto the polyvinylidene difluoride (PVDF) membranes. Next, the membranes were blocked with 4% blocking solution prepared by TBST and bovine serum albumin for 0.5 h on a shaking table. After blocking, the membranes were incubated with specific STAT4 antibody (1:1000, Proteintech, Cat#3028-1-AP) and GAPDH antibody (1:2000, Proteintech, Cat#10494-1-AP) at 4°C overnight. Membranes were washed with TBST and then incubated with 1:2000 secondary antibodies for 1 h, and developed with enhanced chemiluminescence (ECL).

### Cell viability and proliferation assays

After interfering with STAT4 expression, HL60 cells were inoculated into 96-well plates. Three replicate wells were set up for each group, and cell proliferation was detected with the Cell Counting Kit 8 (CCK-8). After co-incubating the CCK-8 reagent with the cells for 1 h, its OD value was measured at 450 nm using microplate reader.

Cell proliferation was assayed using the BeyoClick™ EdU Cell Proliferation Kit. Add 10 µM EdU to fresh DMEM and incubated at 37°C for 2.5 h. The supernatant was removed, 4% paraformaldehyde was added and fixed for 15 min at room temperature, and the fixative was removed and washed with PBS. Add permeabilisation solution (PBS containing 0.3% Triton X-100), incubate at room temperature for 15 min, remove permeabilisation solution and wash with PBS. Staining was done with click reaction solution (composition: Click Reaction Buffer, CuSO_4_, Azide 594, Click Additive Solution) as well as Hoechst 33342, and finally observed under the microscope.

### Cell death assays and cell cycles assays

The Calcein/PI cell death assay kit (Beyotime, C2015M) was used to detect cell death. The Calcein AM (C2015M-1) and PI (C2015M-2) reagents were diluted with buffer, added to Petri dishes, incubated at room temperature and protected from light for 20 min, washed with PBS and placed under a fluorescence microscope for photographs.

Cells were collected from Petri dishes and fixed in 4% paraformaldehyde for 15 min. Add permeable solution (PBS containing 0.3% Triton X-100) for 15 min, and wash with PBS. The PI was diluted with buffer, added to the cells and incubated for 20 min away from light. The cells were washed with PBS and analysed under flow cytometry.

### Statistical analyses

Results are recorded as means ± standard error of the mean for at least three independent experiments and analyzed by R software (Version 4.1.2). The Wilcoxon rank-sum test and the Kruskal–Wallis test were used for the comparison of continuous variables. Pearson analysis was used for the correlation analyses. Data were considered statistically significant as follows: *P<0.05, **P<0.01, ***P<0.001 and ****P<0.0001.

## Results

### Identification of IRGs and TFs in TCGA cohort

**Figure 2:**
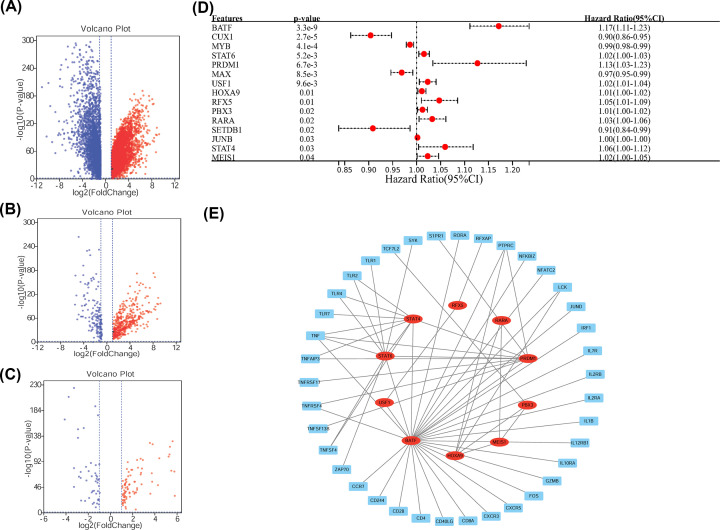
Identification of DEGs (**A**) Volcano plot of DEGs between AML and non-tumor tissues based on the TCGA and GTEx database, The red for up-regulated genes, The blue for down-regulated genes. (**B**) Volcano plot of IRGs. (**C**) Volcano plot of TFs. (**D**) Forest plot demonstrating the univariate Cox model results of 15 OS-related TFs. (**E**) The regulatory network of TFs-IRGs.

We obtained gene expression profiles from TCGA, and after analysis, a total of 10375 DEGs were identified (Figure 2A). Cancer is a multi-step process and requires the activation of transcription factors (TFs) for growth and survival. Many of the TFs reported so far are critical for carcinogenesis [11]. Transcription factors have been reported to play an important role in regulating different aspects of cancer immune function by regulating the expression of immune-related genes [12–14]. Therefore, we identified transcription factors and immune-related genes in the differential genes. About 607 IRGs were selected by matching IRGs to DEGs (Figure 2B). After that, 129 TFs were identified as significantly different by matching TFs to DEGs. Among these, 83 TFs were up-regulated (Figure 2C). To discover the potential prognostic significance of each up-regulated TFs, we performed a univariate Cox hazard regression analysis. The expressions of 15 TFs were found to be significantly associated with AML patient survival (Figure 2D). The DEGs constructed a PPI network based on the STRING database using Cytoscape software to explore the underlying mechanism between the TFs and IRGs (Figure 2E).

### Development of prognostic signature

**Figure 3:**
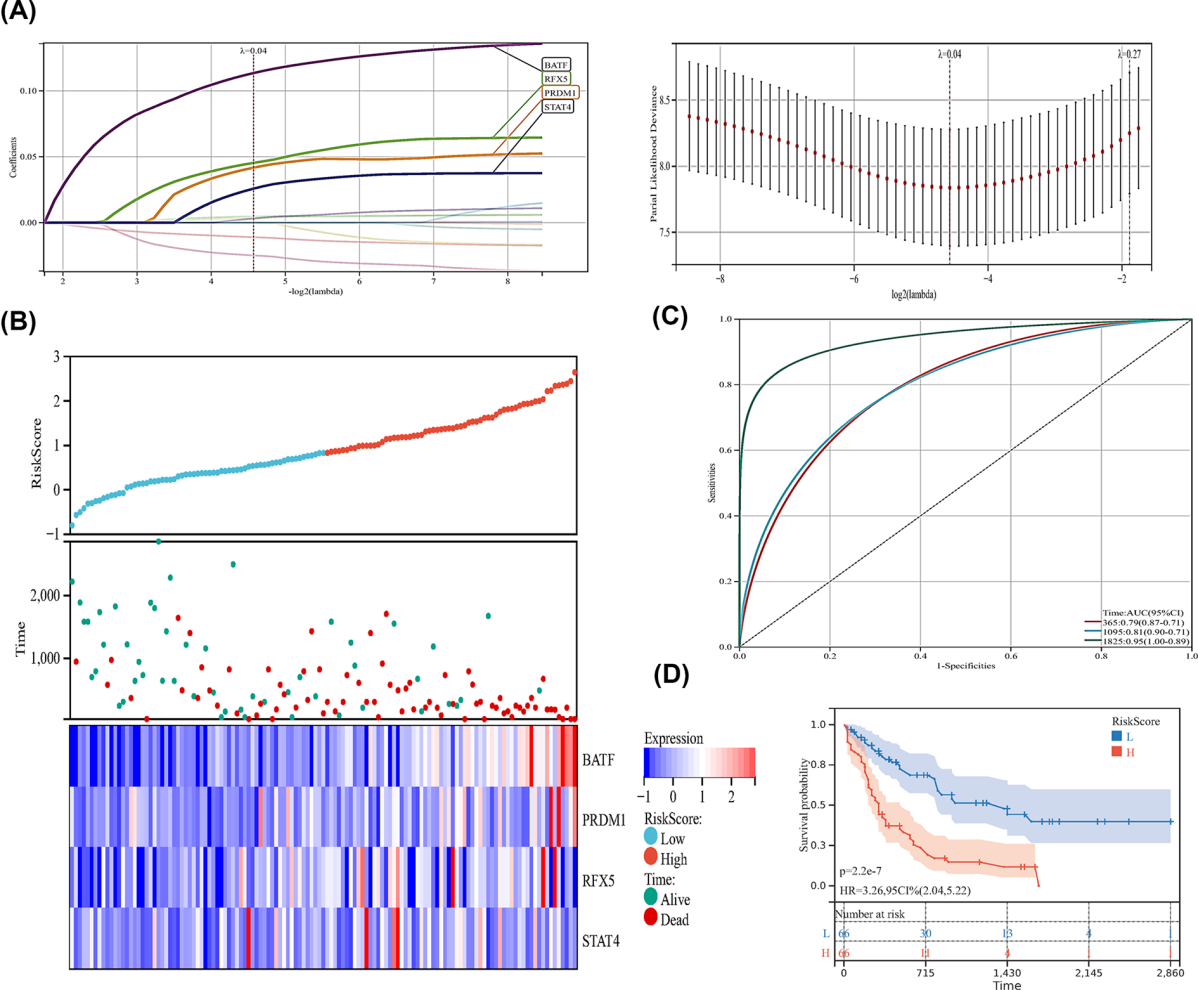
Prognostic analyses for AML patients (**A**) LASSO coefficient profiles of the genes. (**B**) Development of the prognostic signature based on 4-OS-related TFs. (**C**)The AUC curves of the signature for 1, 3 and 5 years. (**D**) OS of AML patients in high- and low-risk groups.

Then, to avoid potential overfitting, LASSO Cox regression analysis was used to select the key OS-related TFs for modeling (Figure 3A). As a result, a total of four TFs (BATF, PRDM1, RFX5, STAT4) were identified and selected to develop a prognostic signature. According to the median risk score, patients were divided into high-risk and low-risk groups (Figure 3B). As the risk score of patients increased, the number of deaths increased (Figure 3B). The heatmap of gene expression between high-risk and low-risk groups in the TCGA cohort was shown (Figure 3B). The AUC of our signature for 1-, 3- and 5-year OS were 0.79, 0.81 and 0.95, respectively, indicating the high predictive capacity of the signature (Figure 3C). The Kaplan–Meier analysis showed that patients in the high-risk group had significantly shorter OS than patients in the low-risk group (Figure 3D).

### DEGs between high-risk and low-risk groups were involved in regulating immune responses

**Figure 4:**
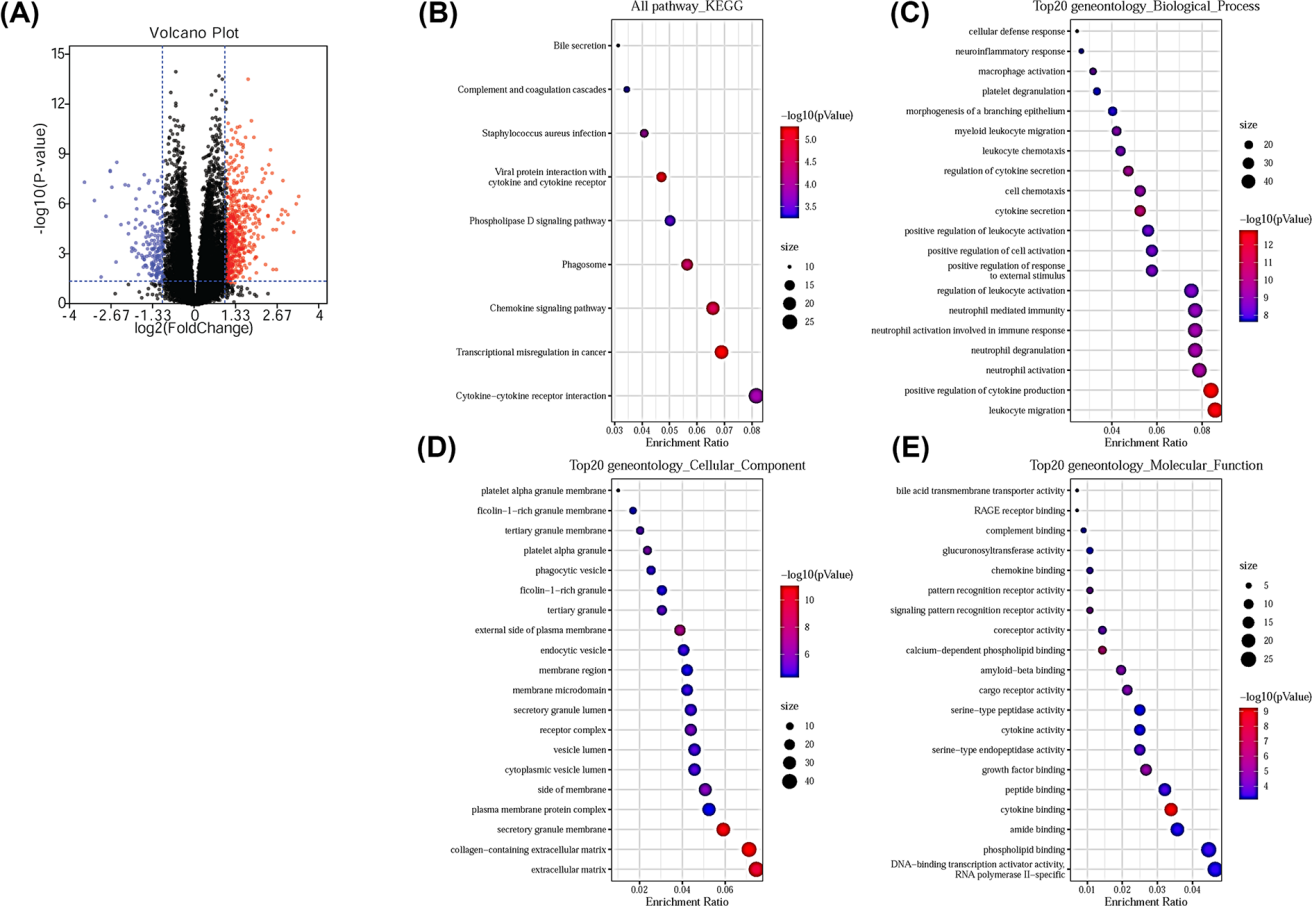
DEGs between the high-risk and the low-risk groups in AML patients (**A**) Volcano plot of the DEGs between the high-risk and the low-risk groups in AML patients, The red for up-regulated genes, The blue for down-regulated genes. (**B**) Significantly enriched KEGG pathway of the DEGs. (**C**) Significantly enriched gene ontology biological process of the DEGs. (**D**) Significantly enriched gene ontology cellular component of the DEGs. (**E**) Significantly enriched gene ontology molecular function of the DEGs.

The results above show the difference in OS between patients in the high-risk and low-risk groups. The two groups were further analyzed to obtain DEG by using the limma package, 499 genes were up-regulated and 179 genes were down-regulated (Figure 4A). The DEGs existed in the KEGG pathway (Figure 4B), biological processes (Figure 4C), cellular component (Figure 4D) and molecular function (Figure 4D) related to immune-related biological processes and activities such as cytokine–cytokine receptor interactions, leukocyte migration and cytokine production. These data suggest that these DEGs may play a role in regulating the immune response in the progression of AML.

### A high level of Tregs in the high-risk group was associated with STAT4

**Figure 5:**
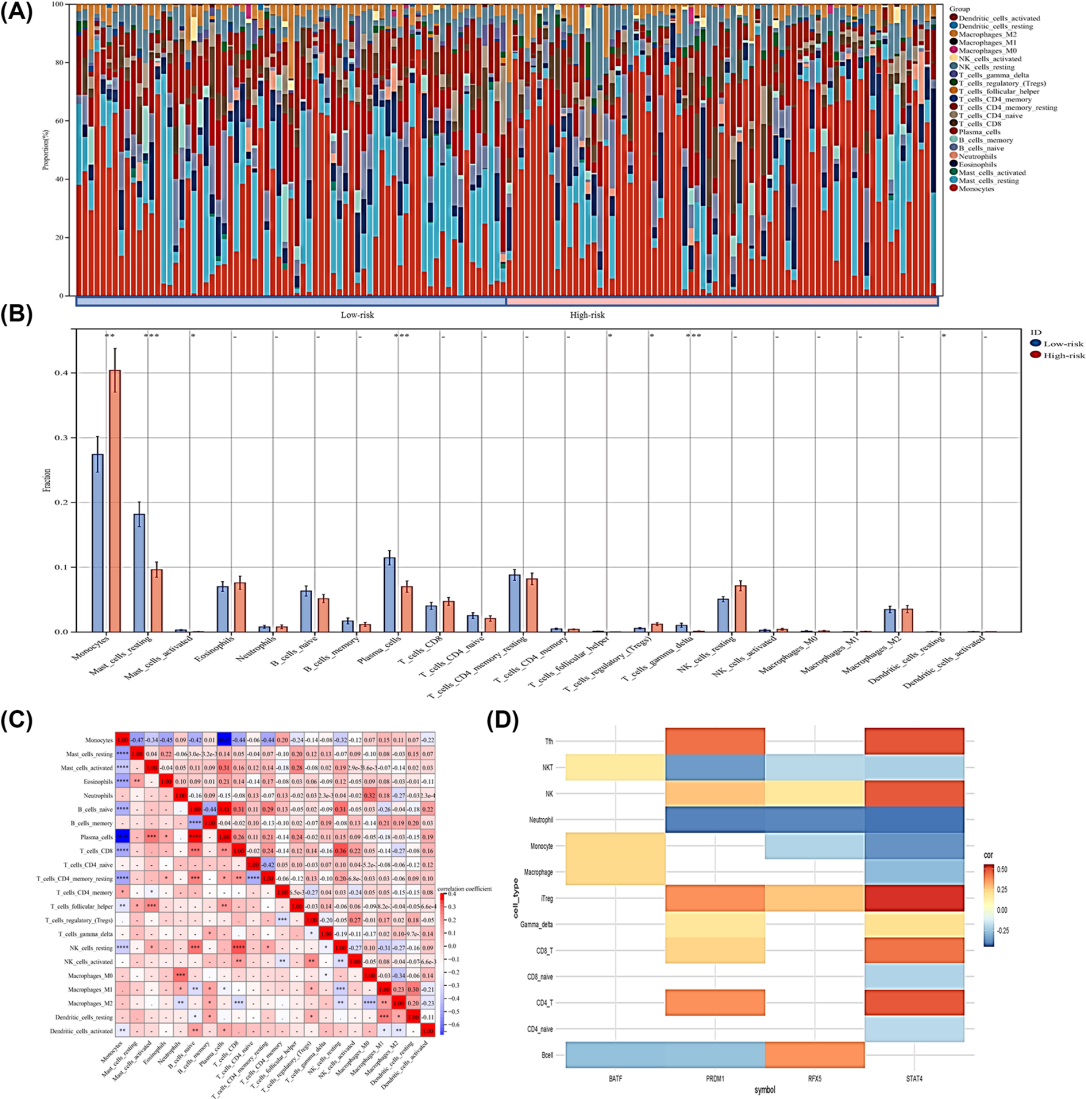
Tumor-infiltrating immune cell analysis of the high- and low-risk groups (**A**) Immune cell profiles in AML samples. (**B**) Differential immune cell type expression was observed between the high and low-risk groups. The red color represents the high-risk score group while the blue color represents the low-risk score group. (**C**) Pearson correlation between immune cells. (**D**) The correlation between genes and each tumor-infiltrating immune cell type. The correlation was performed by using Pearson correlation analysis.

To determine the relationship between the TFs and the immune infiltrating cells, we used the CIBERSORT algorithm to estimate the difference between the low-risk and high-risk groups. A barplot shows the proportion of 22 kinds of immune infiltrating cells in AML cases (Figure 5A). We found that the high-risk group was positively related to Tregs, which are related to immunosuppression, and are negatively related to γΔT cells (Figure 5B), which are related to strong cytotoxic and kill a broad range of tumor cells [15]. We performed Spearman correlation analyses to explore the relationships between immune infiltrating cells (Figure 5C) and TFs (Figure 5D). Significant results are marked with color for the degree of correlation, the results showed a significant correlation between these STAT4 and Tregs (Figure 5D), and STAT4 may be the main contributor to the level of Tregs.

### Validation of prognosis-related genes in external AML cohorts

**Figure 6:**
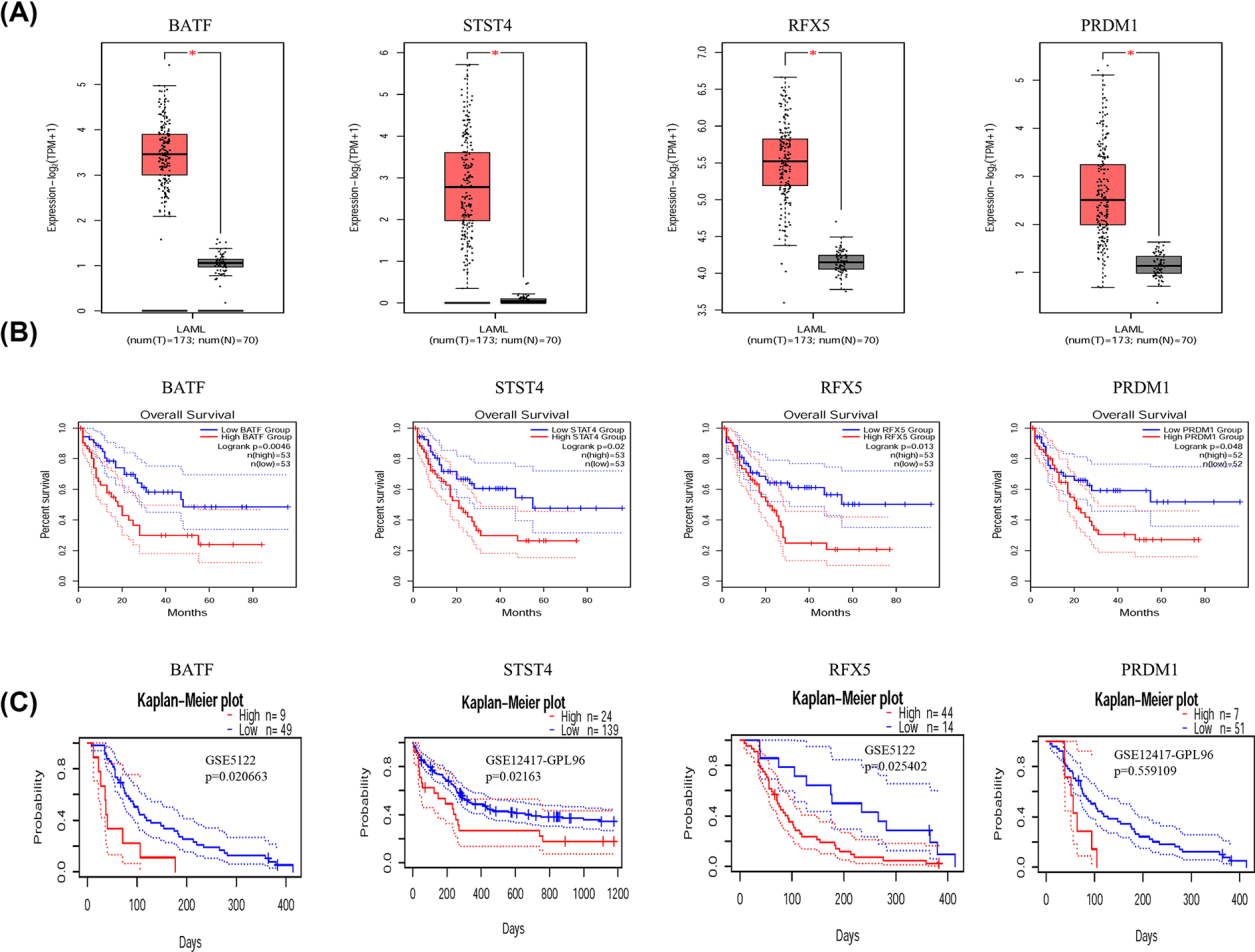
Validation of genes expressions of four risk genes (**A,B**) The expression and impact on survival of BATF, STAT4, RFX5 and PRDM1 in AML and normal tissues. (**C**) Survival analysis for BATF, STAT4, RFX5 and PRDM1 in GEO external AML cohorts.

GEPIA was used to validate the expression and impact on survival of these four prognosis-related genes, BATF, STST4, RFX5 and PRDM1 in AML (Figure 6A,B). The effect of genes on patient prognosis was verified by testing the prognosis of patients’ OS in external AML cohorts (GSE5122 and GSE12417). The results confirmed that patients in the gene high expression group had a worse prognosis (Figure 6C).

### Analysis of the target genes regulated by STAT4

**Figure 7:**
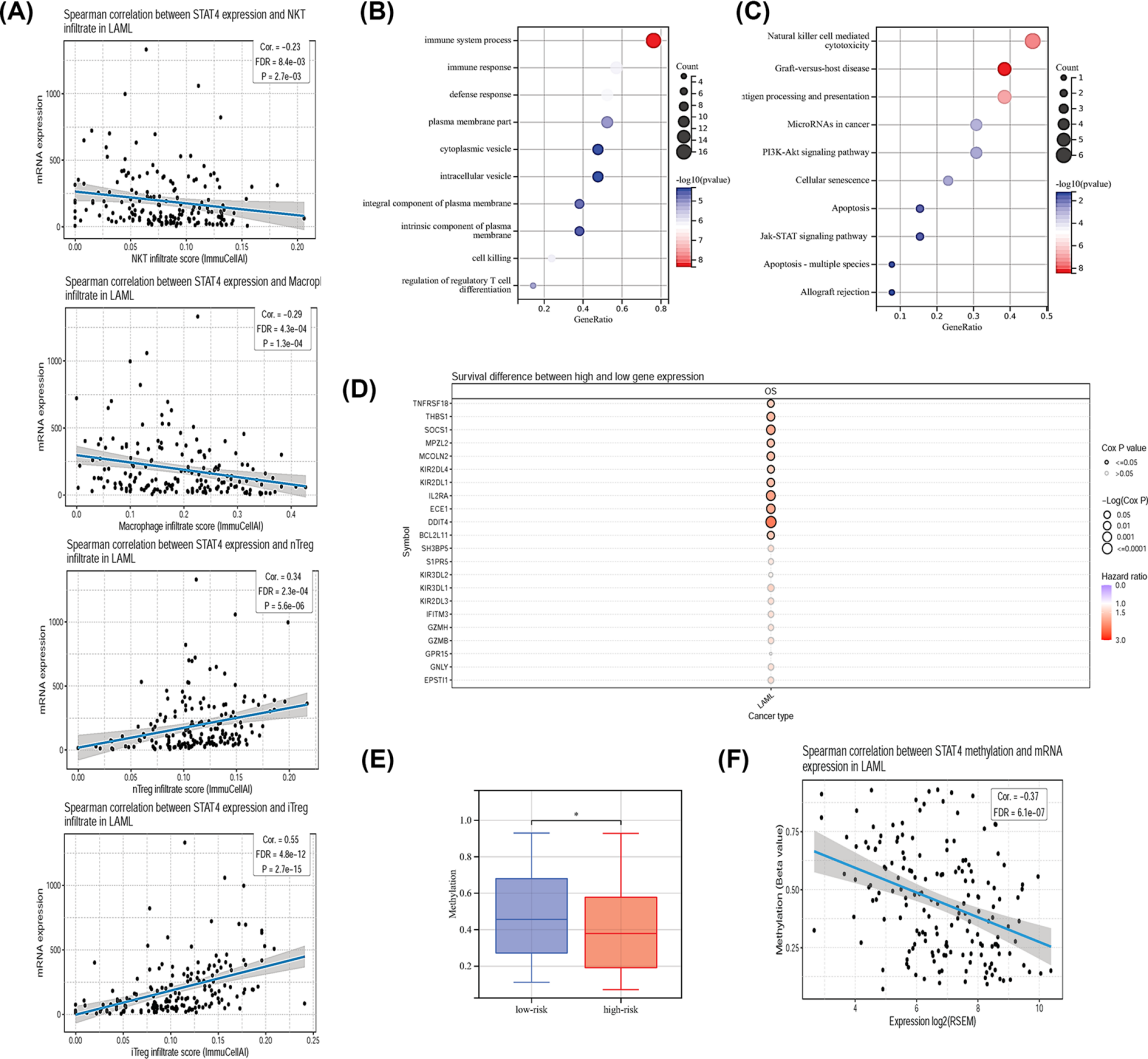
Functional analysis of a group of immune genes regulated by STATA4 (**A**) Pearson correlation of the STAT4 expression and immune cells in AML. (**B,C**) Significantly enriched GO and KEGG of STATA4 transcriptional target genes. (**D**) Characteristics of OS of STATA4 transcriptional target genes. (**E**) Methylation of STAT4 difference between high-risk and low-risk groups. (**F**) Spearman correlation between STAT4 methylation and mRNA expression in AML.

The correlation test between STAT4 expression and immune cells is presented, and it was found that macrophage and NKT were negatively correlated with STAT4 expression, while inducible Treg (iTreg) was positively correlated with STAT4 expression (Figure 7A). The above results suggest that STATA4 affects immunity, and the downstream target genes of STATA4 will be identified next. We obtained a set of immune genes regulated by STATA4 through the ChEA3 website, which integrates a collection of gene set libraries generated from multiple sources including TF-gene co-expression and TF-target associations [16], combined with co-expression analysis to identify possible transcriptional target genes of STATA4. The results of the GO (Figure 7B) and KEGG (Figure 7C) pathway enrichment analysis of target genes regulated by STAT4 demonstrate their involvement in immune system processes, cellular senescence, apoptosis-related pathways, PI3K and other signaling pathways. In addition, we performed a survival analysis of these genes showed that most of them were unfavorable for patient survival (Figure 7D). These genes, which are involved in tumor immunity and are unfavorable to patient survival, may be downstream genes of STAT4 affecting patient prognosis.

High STAT4 expression is detrimental to prognosis, however, the reason for its abnormal high expression is not known. Abnormal epigenetic modifications play an important role in the development of AML, and abnormal DNA methylation is one of the important mechanisms [17]. Abnormal DNA methylation causes the activation and up-regulation of certain genes, leading to the development of AML. It is well known that the transcription level of genes is related to the methylation level of promoter regions, to further infer the reason for the high expression of STAT4 in AML the TCGA database was used to analyze the expression of STAT4 and its methylation status of promoter regions. We found that STAT4 methylation expression in the low-risk group was significantly higher than that in the high-risk group (Figure 7E), and Pearson correlation analysis showed that STAT4 DNA methylation was negatively correlated with mRNA expression (Figure 7F). This result suggests that up-regulation of STAT4 expression may be due to higher methylation of the DNA promoter region.

### Validation in clinical tissue samples and down-regulation of STAT4 inhibits AML growth

**Figure 8:**
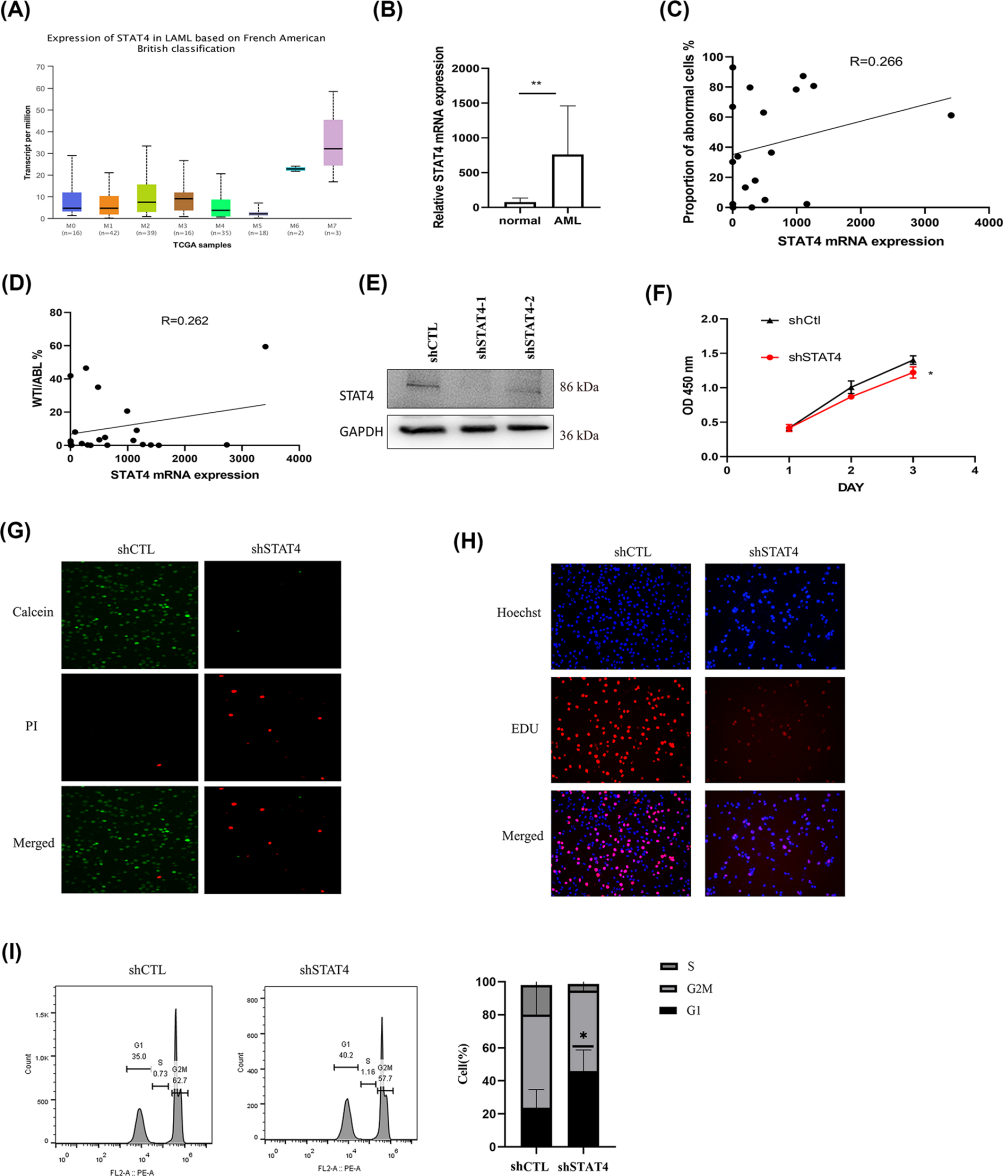
Clinical relevance of STAT4 expression in AML patients and effect on HL60 cells growth (**A**) Expression of STAT4 in AML subtype. (**B**) Differences of STAT4 expression in AML patients and healthy people. (**C,D**) STAT4 is associated with tumor cell ratio and AML recurrence. (**E**) Levels of STAT4 protein in HL60 cells were analyzed by immunoblotting. (**F**) Cell proliferation assays of HL60 cells after transfected as described above, downregulation of STAT4 inhibits the growth of AML cells. (**G**) Cell death assay. (**H**) EDU proliferation assay. (**I**) Cell cycle assay.

Firstly, we focused on the difference of STAT4 gene expression among AML subtype (Figure 8A). To validate the bioinformatics analysis results, 17 AML patient samples were studied to analyze the clinical relevance of STAT4 expression, the higher the expression level of STAT4 in AML compare with normal (Figure 8B). STAT4 expression was positively correlated with the proportion of abnormal cells in AML (Figure 8C) which were similar to the bioinformatics results. WT1 (Wilms tumor 1) /ABL was defined as an indicator of AML recurrence [18]. We observed that STAT4 expression levels positively correlated with WTI/ABL, suggesting that STAT4 promotes AML recurrence (Figure 8D). To study the biological function of STAT4, we established STAT4-silenced cells using a plasmid vector in HL60 cells. Compared with control cells, the experimental results revealed reduction in STAT4 protein expression (Figure 8E). The growth ability of the cells was much lower in STAT4 knockdown cells compared with control cells (Figure 8F). From the cell death assay (Figure 8G), EDU proliferation assay (Figure 8H) and cell cycle assay (Figure 8I), The results showed that that knockdown of STAT4 decreased the proliferative capacity and cell viability of HL-60 cells, and caused cell cycle changes.These results indicated that STAT4 was up-regulated in AML and associated with a poor prognosis, STAT4 promoted AML cells growth.

## Discussion

In the present study, we used the TCGA AML dataset to screen for genes with significant prognostic value and to identify potential prognostic indicators for AML. TFs can play a key role in influencing tumor progression by regulating the transcription of multiple genes [19]. We identified four TFs which were selected for prognostic modeling. BATF is necessary to maintain normal numbers of mature innate lymphoid cells and NK cells in the bone marrow and peripheral tissues [20]. RFX5 promotes cell cycle progression and inhibits apoptosis in hepatocellular carcinoma [21]. Previous research has shown that STAT4 activates different cytokines through the JAK-STAT signaling pathway and plays an important role in a variety of autoimmune and inflammatory diseases [22–25]. We found that STAT4 is most closely related to the prognosis and tumor immunity of AML, so we explored the function and mechanism of STAT4 in AML.

Cancer cells need to evade the host’s antitumor response to survive and metastasize, solid tumors must increase their cell numbers and adapt to their microenvironment [26]. In these respects, members of the STAT family can play a major regulatory role, and they produce different effects by altering gene transcription in effector cells. Seven STAT genes have been identified in the human genome that play an important role in regulating physiological cellular processes and are used to regulate the epigenetics of immune cells [27]. Members of the STAT family are thought to be implicated in the development of human cancers and STAT3 and STAT5 are considered to be carcinogens with important implications for cancer biology. STAT6 expression was increased and found to be an adverse prognosis factor in AML patients, especially those only received chemotherapy treatments [28]. STAT4 appears to have a more limited role in tumor biology. STAT4 is critically involved in the metastasis of ovarian [29] and colon cancers [30]. However, the exact role of STAT4 in cancer remains unclear, STAT4 plays an opposite role in hepatocellular carcinoma [31], breast [32] and gastric [33].

We evaluated the prognostic value of STAT4 in TCGA and GEO datasets. The results of the study showed that high expression of STAT4 is accompanied by lower OS. The results suggest that high STAT4 expression in AML can be used as an independent prognostic indicator for patients with AML. Based on a comprehensive analysis of immune infiltrating cells in the prognostic model, we found that higher levels of immune infiltrating cells in the high-risk group were associated with survival. By combining the correlation between immune infiltrating cells, GSEA and OS with STAT4, we found a significant correlation between STAT4 and tumor immunity, especially for Tregs that exert immunosuppressive effects. STAT4 may act as a transcription factor to regulate immune-related downstream genes to mediate immune responses, but its deeper mechanisms need to be further explored.

In conclusion, the present study’s results suggest that STAT4 is a valid prognostic indicator, and its high expression predicts lower survival and high levels of Tregs, its targeted intervention may be beneficial in treating AML patients.

## Supplementary Material

Supplementary Figure

## Data Availability

In the present study, publicly available datasets were analyzed by NCBI Gene Expression Omnibus (GSE5122 and GSE12417) and the Cancer Genome Atlas (https://portal.gdc.cancer.gov/).

## Abbreviations

AML: acute myelogenous leukemia
DEG: differentially expressed gene
ECL: enhanced chemiluminescence
GEO: Gene Expression Omnibus
IRG: immune-related gene
PVDF: polyvinylidene difluoride
ROC: receiver operating characteristic
TCGA: The Cancer Genome Atlas
TF: transcription factor
Treg: regulatory T cell
WT1: Wilms tumor 1
