# Supplementary figures and images for: Identification and validation of STAT4 as a prognostic biomarker in acute myeloid leukemia
Source: Biosci Rep. 2024 Feb 12;44(2):BSR20231720. doi: 10.1042/BSR20231720 (PMC10861362; doi:10.1042/BSR20231720)

GAPDH

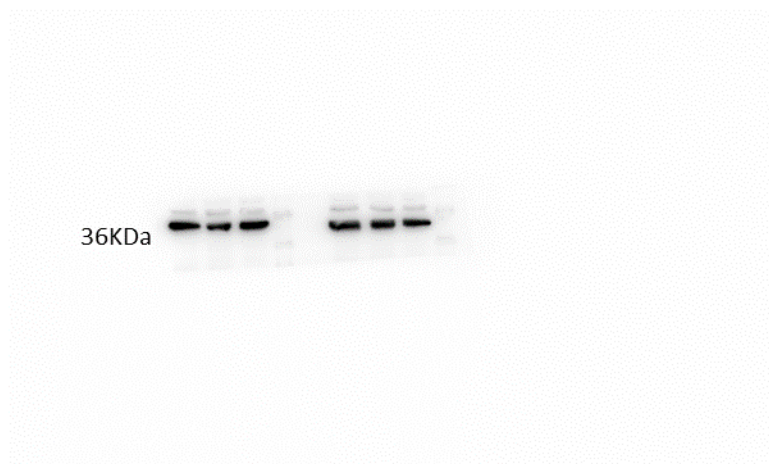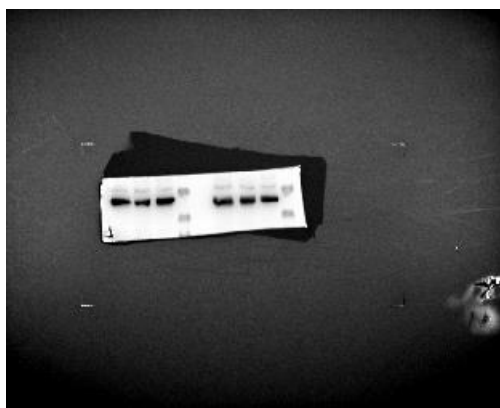

STAT4

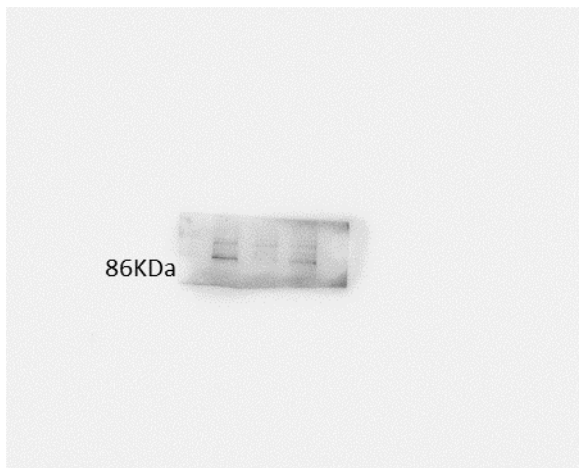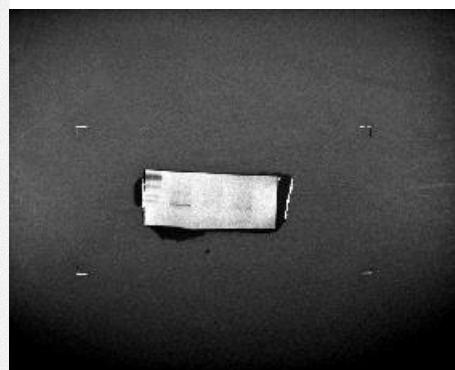

the raw data of western blot

Supplement: Supplementary Figure [file BSR-2023-1720_supp.pdf]
